# COVID-19 and Cancer: Discovery of Difference in Clinical Immune Indexes

**DOI:** 10.1155/2021/8669098

**Published:** 2021-10-18

**Authors:** Xiaojiao Zeng, Xianghu Jiang, Liu Yang, Yunbao Pan, Yirong Li

**Affiliations:** Department of Laboratory Medicine, Zhongnan Hospital of Wuhan University, Wuhan University, Wuhan, China

## Abstract

**Objective:**

This study explored the consistency and differences in the immune cells and cytokines between patients with COVID-19 or cancer. We further analyzed the correlations between the acute inflammation and cancer-related immune disorder.

**Methods:**

This retrospective study involved 167 COVID-19 patients and 218 cancer patients. COVID-19 and cancer were each further divided into two subgroups. Quantitative and qualitative variables were measured by one-way ANOVA and chi-square test, respectively. Herein, we carried out a correlation analysis between immune cells and cytokines and used receiver operating characteristic (ROC) curves to discover the optimal diagnostic index.

**Results:**

COVID-19 and cancers were associated with lymphopenia and high levels of monocytes, neutrophils, IL-6, and IL-10. IL-2 was the optimal indicator to differentiate the two diseases. Compared with respiratory cancer patients, COVID-19 patients had lower levels of IL-2 and higher levels of CD3^+^CD4^+^ T cells and CD19^+^ B cells. In the subgroup analysis, IL-6 was the optimal differential diagnostic parameter that had the ability to identify if COVID-19 patients would be severely affected, and severe COVID-19 patients had lower levels of lymphocyte subsets (CD3^+^ T cells, CD3^+^CD4^+^ T cells, CD3^+^CD8^+^T cells, and CD19^+^ B cells) and CD16^+^CD56^+^ NK cells and higher level of neutrophils. There were significant differences in the levels of CD3^+^CD4^+^ T cells and CD19^+^ B cells between T_1-2_ and T_3-4_ stages as well as IL-2 and CD19^+^ B cells between N_0-1_ and N_2-3_ stages while no significant differences between the metastatic and nonmetastatic cancer patients. Additionally, there were higher correlations between IL-2 and IL-4, TNF-*α* and IL-2, TNF-*α* and IL-4, TNF-*α* and IFN-*γ*, and CD16^+^CD56^+^NK cells and various subsets of T cells in COVID-19 patients. There was a higher correlation between CD3^+^CD4^+^ T cells and CD19^+^ B cells in cancer patients.

**Conclusion:**

Inflammation associated with COVID-19 or cancer had effects on patients' outcomes. Accompanied by changes in immune cells and cytokines, there were consistencies, differences, and satisfactory correlations between patients with COVID-19 and those with cancers.

## 1. Introduction

A new coronavirus appeared in 2019 after two major infectious disease pandemics caused by the severe acute respiratory syndrome coronavirus (SARS-CoV) and the Middle East respiratory syndrome coronavirus (MERS-CoV) in 2002 and 2012, respectively. The virus was named SARS-CoV-2 by the Coronaviridae Study Group (CSG) of the International Committee on Taxonomy of Viruses [1]. Coronavirus disease 2019 (COVID-19) caused by SARS-CoV-2 has a wider and deeper impact and has been declared a Public Health Emergency of International Concern (PHEIC) by the World Health Organization (WHO). There were more than 50 million confirmed cases and more than 1 million deaths by November 2020, and the virus remains a threat to human health [2]. After SARS-CoV-2 infection, the body activates innate and adaptive immunity to implement an immune response, which results in distinct heterogeneity of COVID-19 by involving a series of physiological and pathological mechanisms, such as mild respiratory symptoms and severe respiratory diseases. Acute inflammation is critical for the regulation of tissue repair, regeneration, and homeostasis. In patients with severe COVID-19, activated immune cells produce various cytokines, and then, cytokines act on immune cells and create an amplified inflammatory cascade. Lung injury and death are mainly induced by an excessive inflammatory response [3]. Lymphocyte and macrophage infiltration into the lung parenchyma often occurs in COVID-19 patients [4].

Cancer, the enemy of mankind, is always harmful to human health. The incidence and mortality of cancer are rapidly increasing around the world. According to the GLOBOCAN 2020 report by the International Agency for Research on Cancer, it was estimated that there were approximately 19.3 million new cancer cases and nearly 10 million cancer deaths worldwide in 2020 [5]. In past decades, there was interest in studying the immune-inflammatory response that occurs in cancer. Infectious (*Helicobacter pylori*, human papillomavirus, and hepatitis B virus) and noninfectious stimulation (obesity, smoking, and alcohol consumption) can lead to the proliferation and activation of immune cells and cytokines and the formation of an inflammatory microenvironment. The stimulation of chronic inflammation easily promotes the occurrence, progression, and metastasis of cancer and cancer-related immune disorder persists and forms the tumor microenvironment [6–8].

Numerous studies have shown that there are significant changes in immune cells and cytokines during the occurrence and development of COVID-19 and cancer. Tan and Yang [9] revealed that SARS-CoV-2 can activate various immune cells (T cells, B cells, macrophages, and natural killer (NK) cells) and cytokines (interleukin- (IL-) 2, IL-4, IL-6, interferon- (IFN-) *γ*, and tumor necrosis factor- (TNF-) *α*), which can lead to excessive inflammation and pulmonary immunopathology. Dranoff's study in 2004 found that there were complex immune cells and cytokines in the tumor microenvironment, and the interaction between them played a decisive role in the antitumor immune response [10].

In this study, we analyzed the immune cells and cytokines between COVID-19 and cancer patients to explore the consistency, difference, and differential diagnostic efficiency of these indexes between the acute inflammation and cancer-related immune disorder. In addition, COVID-19 and cancer patients were each divided into two groups according to the condition of severity and metastasis. We analyzed the immune cells and cytokines of the four subgroups to discover the correlations, influencing factors, and the optimal diagnostic indexes among COVID-19 and cancer patients.

## 2. Materials and Methods

### 2.1. Patients

We retrospectively recruited 167 and 218 patients who were diagnosed with COVID-19 or cancer at the Zhongnan Hospital of Wuhan University from December 2018 to October 2020, respectively. There were 86 male and 81 female patients with COVID-19, with a median age of 58 (range 17-93 years). According to the clinical classification standard of the 7th edition of Guidelines for the Diagnosis and Treatment of COVID-19, those with severe or critical disease were classified as the severe group, while mild and ordinary types were classified as nonsevere groups. There were 51 severe and 116 nonsevere patients. There were 152 males and 66 females with a median age of 57 (range 9-85 years) who had respiratory and nonrespiratory cancers. There were 96 nonmetastatic and 29 metastatic cancer patients, and the status of 93 cancer patients was unknown.

### 2.2. Inclusion Criteria and Enrollment

The inclusion criteria in this study were (1) patients with definite diagnosis of COVID-19 and solid cancers and (2) patients with complete records of immune cells and cytokines. Exclusion criteria were as follows: (1) patients with both COVID-19 and cancers, (2) patients with hematological malignancy, and (3) patients with incomplete medical records.

### 2.3. Physical Examination and Hematological Data

Peripheral blood cells, such as neutrophils, lymphocytes, and monocytes, were analyzed with a Beckman Coulter DxH 800 automated blood analyzer according to the manufacturer's instructions (Beckman, California, USA). Serum cytokines, including IL-2, IL-4, IL-6, IL-10, TNF-*α*, and IFN-*γ*, were analyzed by BD FACSCalibur flow cytometry according to the manufacturer's instructions. Peripheral absolute cell counts of CD3^+^ T cells, CD3^+^CD4^+^ T cells, CD3^+^CD8^+^ T cells, CD19^+^ B cells, and CD16^+^CD56^+^natural killer (NK) cells were obtained by flow cytometry according to the manufacturer's instructions.

### 2.4. Statistical Analysis

The statistical analyses were conducted by IBM SPSS version 22.0 software (SPSS, Chicago, IL). Quantitative and qualitative variables were measured by one-way ANOVA and chi-square test, respectively. Pairwise comparisons and correlation analyses between groups were plotted by GraphPad Prism v7.0 software. The receiver operating characteristic (ROC) curve was applied to assess the diagnostic efficiency of various immune indicators. A *p* value < 0.05 was considered statistically significant.

## 3. Results

### 3.1. Baseline Characteristics between COVID-19 and Cancer Patients

The patients recruited in our study consisted of those with COVID-19 or cancer of various types. The general parameters of the patients were shown in Table 1. Cancer was common in men and younger patients, with respiratory cancers being the most common. There were significant differences between metastatic and nonmetastatic cancer patients as well as between severe and nonsevere COVID-19 patients. Moreover, most cytokines and immune cells were significantly different, including IL-6, IL-10, CD3^+^ T cells, CD3^+^CD4^+^ T cells, 4/8 ratio, and CD19^+^ B cells.

### 3.2. Variance Analysis of Immune Cells and Cytokines in Different Groups

**Figure 1:**
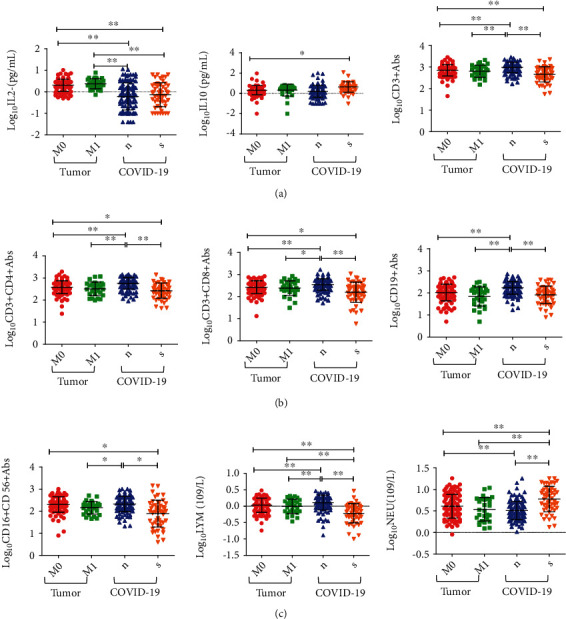
Analysis of immune cells and cytokines in the four subgroups of patients with COVID-19 or cancer. *n*: nonsevere; *s*: severe. (a) IL-2 (left), IL-10 (middle), and CD3^+^ Abs (right). (b) CD3^+^CD4^+^ Abs (left), CD3^+^CD8^+^ Abs (middle), and CD19^+^ Abs (right). (c) CD16^+^CD56^+^ Abs (left), LYM (middle), and NEU (right).

COVID-19 and cancer patients were each further divided into two subgroups, for a total of four subgroups, and the logarithmic levels of immune cells and cytokines were displayed in Figure S1. There were 96 nonmetastatic and 29 metastatic cancer patients, and 116 nonsevere and 51 severe COVID-19 patients, respectively (Table 1). Immune cells and cytokines of COVID-19 and cancer patients were analyzed by one-way ANOVA (Figure 1). The results showed that there were significant differences in the levels of IL-2, IL-10, absolute count of lymphocyte subsets (CD3^+^ T cells, CD3^+^CD4^+^ T cells, CD3^+^CD8^+^ T cells, and CD19^+^ B cells), CD16^+^CD56^+^ NK cells, and neutrophils between patents with COVID-19 and cancers. Moreover, immune cell levels were statistically different between COVID-19 subgroups, including lymphocyte subsets, NK cells, and neutrophils, whereas there was no significant difference between metastatic and nonmetastatic cancer subgroups (Figure 1). Moreover, we further analyzed immune cells and cytokines in different T stages, N stages, and differentiation of cancers (Table S1). There were significant differences in the levels of CD3^+^CD4^+^ T cells and CD19^+^ B cells between T_1-2_ and T_3-4_ stages as well as IL-2 and CD19^+^ B cells between N_0-1_ and N_2-3_ stages (Figure S2).

**Figure 2:**
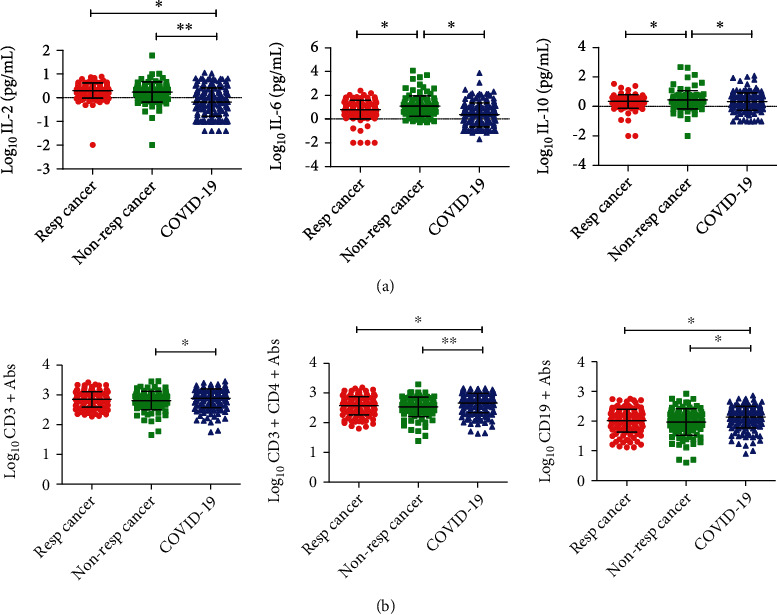
Analysis of immune cells and cytokines in three groups of patients with COVID-19 or cancer. Resp: respiratory cancer; Non-resp: nonrespiratory cancer. (a) IL-2 (left), IL-6 (middle), and IL-10 (right). (b) CD3^+^ Abs (left), CD3^+^CD4^+^Abs (middle), and CD19^+^ Abs (right).

There were 121 patients with respiratory cancers and 97 patients with nonrespiratory cancers (Table S1). And there were significant differences in the levels of NK cells and neutrophils between squamous carcinoma and adenocarcinoma in patients with respiratory cancers as well as NK cells between patients with distant and nondistant metastatic respiratory cancers (Figure S2). A further analysis was conducted of immune cells and cytokines among patients with COVID-19 or cancer. The results suggested that there were significant differences in the levels of IL-2, IL-6, IL-10, CD3^+^ T cells, CD3^+^CD4^+^T cells, and CD19^+^ B cells (Figure 2).

### 3.3. Correlation Analysis of Immune Cells and Cytokines

**Figure 3:**
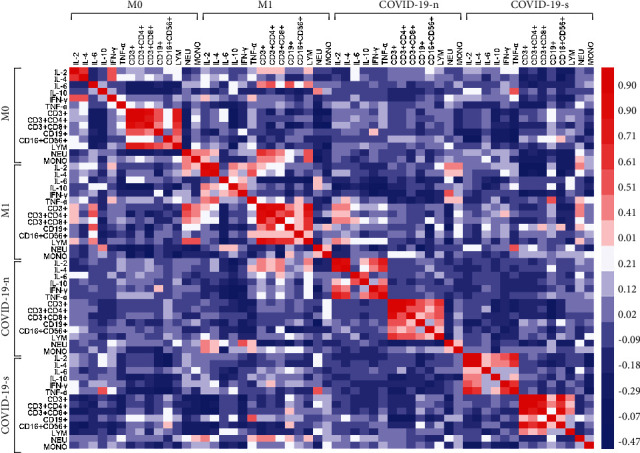
Heat maps for correlation coefficients of patients with COVID-19 or cancer. M0: nonmetastatic cancer patients; M1: metastatic cancer patients; COVID-19-n nonsevere COVID-19 patients; COVID-19-s: severe COVID-19 patients.

**Figure 4:**
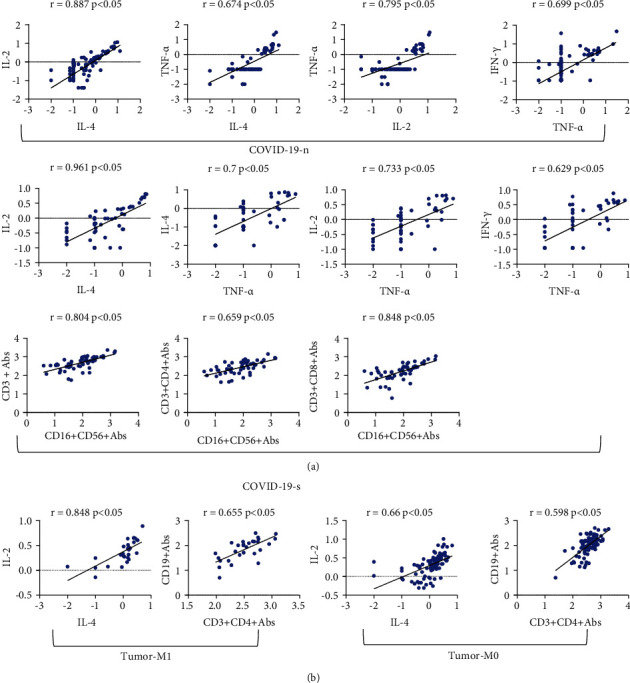
The correlation analysis of the four subgroups of patients with COVID-19 or cancer. (a) COVID-19 patients. (b) Cancer patients.

A correlation analysis was performed on immune cells and cytokines among patients with COVID-19 or cancer (Figure 3). The results suggested that there was a satisfactory correlation among most of these inflammatory markers, and the correlation analysis was plotted (Figure 4). In the severe COVID-19 group, the correlation coefficients between IL-4 and IL-2, CD16^+^CD56^+^ NK cells and CD3^+^ T cells, CD16^+^CD56^+^ NK cells and CD3^+^CD4^+^ T cells, CD16^+^CD56^+^ NK cells and CD3^+^CD8^+^ T cells, TNF-*α* and IL-2, TNF-*α* and IL-4, and TNF-*α* and IFN-*γ* were 0.961, 0.804, 0.659, 0.848, 0.733, 0.7, and 0.629, respectively. In the nonsevere COVID-19 group, the correlation coefficients of IL-4 and IL-2, TNF-*α* and IL-2, TNF-*α* and IFN-*γ*, and TNF-*α* and IL-4 were 0.887, 0.795, 0.699, and 0.674, respectively. In the metastatic group, the correlation coefficient between IL-4 and IL-2, and CD3^+^CD4^+^ T cells and CD19^+^ B cells were 0.848 and 0.655, respectively. Additionally, the correlation coefficients were 0.66 and 0.598 in the nonmetastatic group for two pairs of indexes, respectively. Moreover, the correlation coefficients for IL-4 and IL-2, IL-4 and TNF-*α*, and NK cells and T cells were the lowest in nonmetastatic patients, followed by metastatic and nonsevere COVID-19 patients, and were the highest in severe COVID-19 patients. The correlation coefficients for TNF-*α* and IL-2, and TNF-*α* and IFN-*γ* were higher in the COVID-19 group than that in the cancer group, while the correlation coefficients between CD3^+^CD4^+^ T cells and CD19^+^ B cells were higher in the cancer group than that in the COVID-19 group (Figure 3).

**Figure 5:**
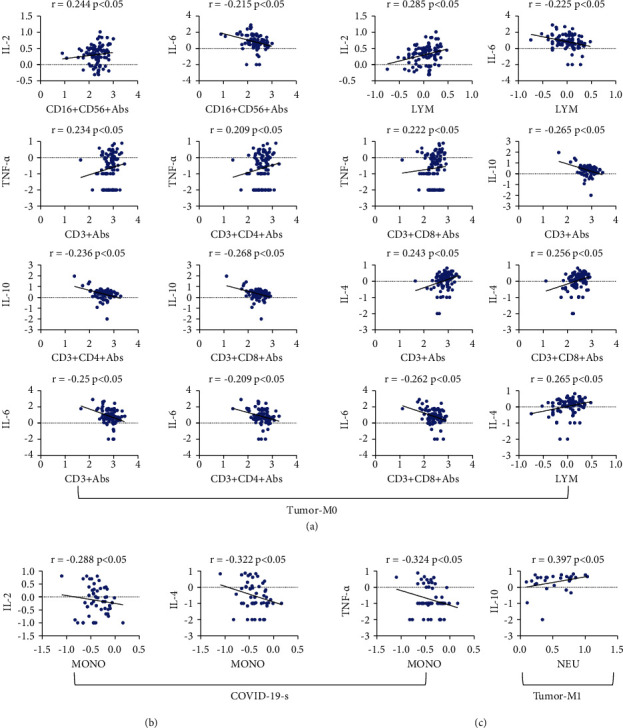
The correlation analysis between immune cells and cytokines of patients with COVID-19 or cancer. (a) Nonmetastatic cancer patients. (b) Severe COVID-19 patients. (c) Metastatic cancer patients.

In addition, there was a significant correlation between immune cells and cytokines (Figure 5). Monocytes were negatively correlated with IL-2, IL-4, and TNF-*α* in the COVID-19 severe group. In the metastatic group, neutrophils were positively correlated with IL-10. In the nonmetastatic group, NK cells and lymphocytes were positively correlated with IL-2 and negatively correlated with IL-6, and T cells were positively correlated with IL-4 and TNF-*α* and negatively correlated with IL-6 and IL-10.

### 3.4. ROC Curve Analysis of Immune Cells and Cytokines

**Figure 6:**
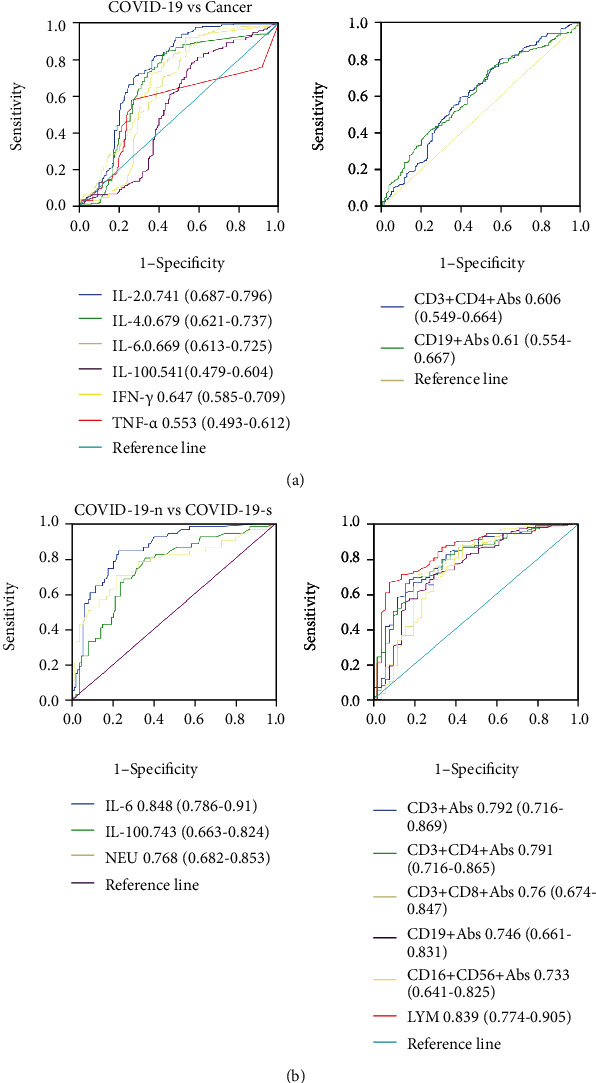
ROC curve analysis of immune cells and cytokines. (a) Between COVID-19 and cancer patients and (b) between severe and nonsevere COVID-19 patients.

We used ROC curves to explore the ability of immune cells and cytokines to differentiate between COVID-19 and cancers (Figure 6(a)). The area under the ROC curve (AUC) for IL-2 (0.741, 0.687-0.796) was larger than that for the other indexes, indicating that IL-2 was the most optimal differential diagnostic value between the two diseases. The AUC of the cytokines IL-4, IL-6, IL-10, IFN-*γ*, and TNF-*α* was 0.679, 0.669, 0.541, 0.647, and 0.553, respectively. The AUCs of the CD3^+^CD4^+^ T and CD19^+^ B immune cells were 0.606 and 0.61, respectively. We further investigated the ability of immune indexes to differentiate between severe and nonsevere COVID-19 (Figure 6(b)). The AUCs of immune indexes such as IL-6, IL-10, neutrophils, CD3^+^ T cells, CD3^+^CD4^+^ T cells, CD3^+^CD8^+^ T cells, CD19^+^ B cells, CD16^+^CD56^+^ NK cells, and lymphocytes were 0.848, 0.743, 0.768, 0.792, 0.791, 0.76, 0.746, 0.733, and 0.839, respectively. These results suggested that there was more optimal differential diagnostic value for IL-6 and lymphocytes between the two groups of COVID-19 patients. However, they exhibited poor diagnostic efficiency in differentiating distant metastatic patients from nonmetastatic patients (Figure S3).

## 4. Discussion

Most studies have shown that COVID-19 is often accompanied by lymphopenia, and high levels of neutrophils and mononuclear macrophages in patients. Cytokine storms with significant increases in IL-6 and IL-10 often occur in patients with severe COVID-19 and may play an important role in the development of lymphopenia, high neutrophils, and high mononuclear macrophages [11–13]. Giamarellos-Bourboulis et al. [14] reported that immune dysregulation in cases of severe COVID-19 was mainly characterized by low expression of IL-6-mediated human leukocyte antigen D-related (HLA-DR) and a decrease in lymphocytes and NK cells. Jamilloux et al. [15] found that the type I IFN response was prolonged or decreased in COVID-19 patients, the innate and adaptive immune responses were suppressed, and both NK cells and lymphocyte subsets were reduced, which indicates that SARS-CoV-2 is not subject to immune control.

An excessive inflammatory response is significantly correlated with poor prognosis in COVID-19 patients. Moreover, Li et al. [16] showed that IL-2, IL-4, IFN-*γ*, and TNF-*α* levels were not significantly different between deceased patients and survivors of COVID-19, and the absolute counts of CD3^+^ T cells, CD3^+^ CD4^+^ T cells, and CD3^+^CD8^+^ T cells in the deceased group were always at a low level. Wu et al. [17] showed that there were no significant differences in IL-6, IFN-*γ*, TNF-*α*, or lymphocyte levels between patients with mild and moderate COVID-19, while the IL-10 level was significantly increased, and the neutrophil level was significantly decreased in the moderate group.

In this study, there were no significant differences in cytokines between the severe and nonsevere COVID-19 groups. In accordance with the conclusion of previous studies, the neutrophils were significantly elevated, and the absolute counts of CD3^+^ T cells, CD3^+^CD4^+^ T cells, CD3^+^CD8^+^ T cells, CD19^+^ B cells, and NK cells were significantly decreased in patients with severe COVID-19. Moreover, ROC curve analysis suggested that IL-6 was the optimal diagnostic index to distinguish severe and nonsevere COVID-19 in patients.

Currently, many studies have proved the relationship between chronic inflammation and the occurrence and development of cancers. Our previous studies also reported the relationship between immune cells and prognosis of cancer [18]. Neutrophils and monocytes can induce immune tolerance, distant metastasis, chemotherapy resistance, and cancer progression by forming tumor-associated neutrophils (TAN) and tumor-associated macrophages (TAM). The effective immunity of cancer mainly depends on the function of NK cells and CD3^+^CD8^+^ T cells, as cytotoxic lymphocytes of the innate and adaptive immune system, respectively [19–21]. Toyoshima et al. [22] reported that high levels of IL-6 can interfere with type-I IFN signals in the immune system and is accompanied by low expression of major histocompatibility complex class I (MHC I) molecules, which can then weaken the antitumor effect of CD3^+^CD8^+^ T cells and promote tumor growth, development, and distant colonization. IL-10, an anti-inflammatory cytokine, protects the body from damage caused by immune overreaction, while IL-10 in cancer has two opposite effects. A high level of IL-10 has an immunosuppressive effect that facilitates the immune escape of cancer cells, but IL-10 also has an antitumor effect by increasing the infiltration of CD3+CD8+ T cells and the production of IFN-*γ* in tissues, which may be related to the fact that IL-10 targets different cells (myeloid and T cells) in different cancers or that T cells respond differently to IL-10 at different effective stages [23].

These studies showed that both COVID-19 and cancer have the phenomenon of interference in the type-I IFN response, immunosuppression, and high levels of cytokines, such as IL-6 and IL-10. Our results were consistent with these conclusions. When we analyzed the immune cells and cytokines between COVID-19 and cancer patients, we discovered that there were similar changes in these biomarkers, such as low lymphocytes, high monocytes, high neutrophils, high IL-6, and high IL-10. The ROC curve suggested that IL-2 was the optimal diagnostic index for both diseases. We further compared the immune cells and cytokines in patients with COVID-19 or respiratory cancers. The results showed that there was no significant difference in IL-6 and IL-10 between the two groups, while COVID-19 patients had lower levels of IL-2 and higher levels of CD3^+^CD4^+^ T cells and CD19^+^ B cells. Moreover, the interaction between cancer and inflammation is regulated through a complex network. The inflammatory response may be varied at different stages of cancer development. Our study suggested that cancer patients in T_3-4_ stages had lower levels of CD3^+^CD4^+^ T cells and CD19^+^ B cells, and patients in N_2-3_ stages had lower levels of CD19^+^ B cells and higher levels of IL-2. In addition, we conducted a correlation analysis of these inflammatory indexes in COVID-19 and cancers, and the results suggested that there was a higher correlation between IL-2 and IL-4, NK cells and T cells in COVID-19 patients, and a higher correlation between CD3^+^CD4^+^ T cells and CD19^+^ B cells in cancer patients.

Luo et al. [24] revealed that multiple elevated cytokines were associated with poor prognosis of severe COVID-19 patients, including IL-2, IL-4, IL-6, IL-10, IFN-*γ*, and TNF-*α*. With the same cytokine receptor *γ* chain, IL-2 and IL-4 together regulate cell differentiation, promote the formation of immune cells, improve the killing activity of cytotoxic T lymphocyte (CTL) and NK cells, and play an important role in inflammation and cancers [25]. Our study showed that there was a good correlation between IL-2 and IL-4 in patients with COVID-19 or cancers.

TNF-*α* can guide circulating monocytes to the site of injury so that they can differentiate into mature macrophages [6]. Cytokines secreted by macrophages can activate NK cells, and IFN-*γ* produced by NK cells acts on alveolar macrophages to amplify the inflammatory response [26]. Karki et al. [27] reported that the synergistic effects of TNF-*α* and IFN-*γ* in COVID-19 patients can induce various types of cell death and tissue damage and result in a poor prognosis. In patients with severe COVID-19, we found that TNF-*α* was satisfactorily correlated with IL-2, IL-4, and IFN-*γ*, and monocytes were negatively correlated with IL-2, IL-4, and TNF-*α*. In nonmetastatic cancer patients, T cells were negatively correlated with IL-6 and IL-10 and were positively correlated with IL-4 and TNF-*α*.

Although we conducted an analysis of different immune cells and cytokines between COVID-19 and cancers, there were some deficiencies in our study. The examination of cytokines was carried out in 2018, and there was incomplete case information for many cancer patients. Thus, only 29 metastatic patients were recruited, and they were accompanied by 93 cases with unknown metastatic status in this study. In addition, this was a single-center retrospective study. We require data from a larger and multicenter cohort to better assess the changes in immune response after acute infection and cancer-related immune disorder.

In conclusion, both COVID-19 and cancers were associated with lymphopenia and high levels of monocytes, neutrophils, IL-6, and IL-10. IL-2 was the optimal indicator to differentiate between the COVID-19 and cancer-related immune disorder. In comparing patients with respiratory cancers, COVID-19 patients had lower levels of IL-2 and higher levels of CD3^+^CD4^+^ T cells and CD19^+^ B cells, and no difference in levels of IL-6 and IL-10. In addition, there were higher correlations between IL-2 and IL-4, TNF-*α* and IL-2, TNF-*α* and IL-4, TNF-*α* and IFN-*γ*, and NK cells and T cells in COVID-19 patients, and there was a higher correlation between CD3^+^CD4^+^ T cells and CD19 + T cells in cancer patients. Moreover, there were lower levels of lymphocyte subsets (CD3^+^ T cells, CD3^+^CD4^+^ T cells, CD3^+^CD8^+^ T cells, and CD19^+^ B cells) and NK cells and higher level of neutrophils in severe COVID-19 patients, and IL-6 exhibited the most optimal ability for differential diagnosis between severe and nonsevere COVID-19 patients. As for cancer patients, there were no significant differences in immune cells and cytokines between the metastatic and the nonmetastatic group.

## Figures and Tables

**Table 1:** Baseline parameters between COVID-19 and cancer patients.

Variables	*n*	*p*
Sex		≤0.001
Cancer		
Male	152	
Female	66	
COVID-19		
Male	86	
Female	81	
Age		0.646
Cancer		
<60	120	
≥60	98	
COVID-19		
<60	88	
≥60	79	
COVID-19		≤0.001
Nonsevere	116	
Severe	51	
Cancer type		0.104
Respiratory system	121	
Nonrespiratory system	97	
Cancer distant metastasis		≤0.001
M0	96	
M1	29	
Unknown	93	
IL-2		0.906
Cancer		
Normal	195	
High	23	
COVID-19		
Normal	150	
High	17	
IL-4		0.156
Cancer		
Normal	198	
High	20	
COVID-19		
Normal	144	
High	23	
IL-6		≤0.001
Cancer		
Normal	48	
High	170	
COVID-19		
Normal	83	
High	84	
IL-10		≤0.001
Cancer		
Normal	188	
High	30	
COVID-19		
Normal	118	
High	49	
IFN-*γ*		0.105
Cancer		
Normal	218	
High	0	
COVID-19		
Normal	165	
High	2	
TNF-*α*		0.850
Cancer		
Normal	217	
High	1	
COVID-19		
Normal	166	
High	1	
CD3^+^Abs Cnt		0.028
Cancer		
Normal	102	
Low	116	
COVID-19		
Normal	97	
Low	70	
CD3^+^CD4^+^Abs Cnt		0.003
Cancer		
Normal	126	
Low	92	
COVID-19		
Normal	121	
Low	46	
CD3^+^CD8^+^Abs Cnt		0.109
Cancer		
Normal	79	
Low	139	
COVID-19		
Normal	74	
Low	93	
4/8 ratio		0.014
Cancer		
Normal	110	
High	56	
Low	52	
COVID-19		
Normal	90	
High	56	
Low	21	
CD19^+^Abs Cnt		0.019
Cancer		
Normal	35	
Low	183	
COVID-19		
Normal	43	
Low	124	
CD16^+^CD56^+^Abs Cnt		0.403
Cancer		
Normal	102	
Low	116	
COVID-19		
Normal	71	
Low	96	
LYM		0.625
Cancer		
Normal	111	
High	1	
Low	106	
COVID-19		
Normal	89	
Low	78	
NEU		0.260
Cancer		
Normal	144	
High	53	
Low	21	
COVID-19		
Normal	122	
High	35	
Low	10	
MONO		0.268
Cancer		
Normal	148	
High	65	
Low	5	
COVID-19		
Normal	126	
High	38	
Low	3	

Abbreviations: M: metastasis; CD3^+^Abs: absolute count of CD3^+^T cells; CD3^+^CD4^+^Abs: absolute count of CD3^+^CD4^+^T cells; CD3^+^CD8^+^Abs: absolute count of CD3^+^CD8^+^T cells; CD19^+^Abs: absolute count of CD19^+^B cells; CD16^+^CD56^+^Abs: absolute count of CD16^+^CD56^+^NK cells; LYM: lymphocyte; NEU: neutrophil; MONO: monocyte.

## Data Availability

The original contributions presented in the study are included in the article material. Further inquiries can be directed to the corresponding authors.

## Supplementary Materials

Supplementary 1 Figure S1: heat maps for levels of various immune cells and cytokines in patients with COVID-19 or cancer.

Supplementary 2 Figure S2: analysis of immune cells and cytokines in different stages and differentiations of cancers. (a) Between T_1-2_ and T_3-4_ and (b) between N_0-1_ and N_2-3_ and (c) between squamous carcinoma and adenocarcinoma of respiratory cancers and (d) between M0 and M1 of respiratory and nonrespiratory cancers. (a) CD3^+^CD4^+^ Abs (left), CD19^+^ Abs (middle), LYM (right). (b) IL-2 (left), CD19^+^ Abs (right). (c) CD16^+^CD56^+^ Abs (left), NEU (right). (d) IL-6 (left), TNF-*α* (middle), CD16^+^CD56^+^ Abs (right). (e) Sex variables in different TNM stages of cancer patients.

Supplementary 3 Figure S3: ROC curve analysis of immune cells and cytokines between metastatic and nonmetastatic patients.

Supplementary 4 Table S1: stages and differentiation types of cancers.
