# Supplementary figures and images for: COVID-19 and Cancer: Discovery of Difference in Clinical Immune Indexes
Source: J Immunol Res. 2021 Oct 18;2021:8669098. doi: 10.1155/2021/8669098 (PMC8546403; doi:10.1155/2021/8669098)

Fig. S1

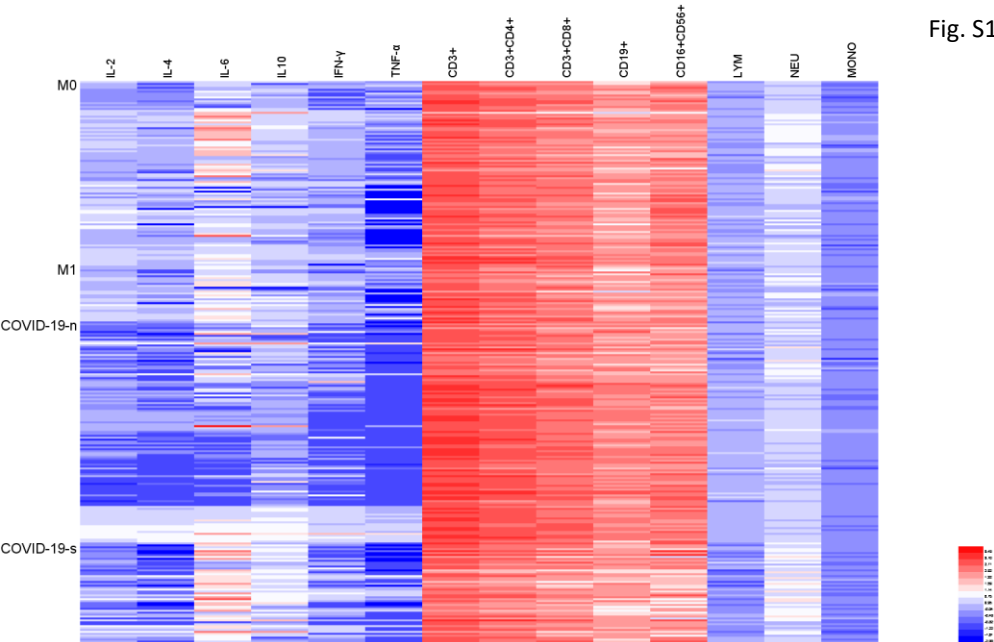

Supplement: Supplementary 1 — Figure S1: heat maps for levels of various immune cells and cytokines in patients with COVID-19 or cancer. [file 8669098.f1.pdf]

Fig.S2

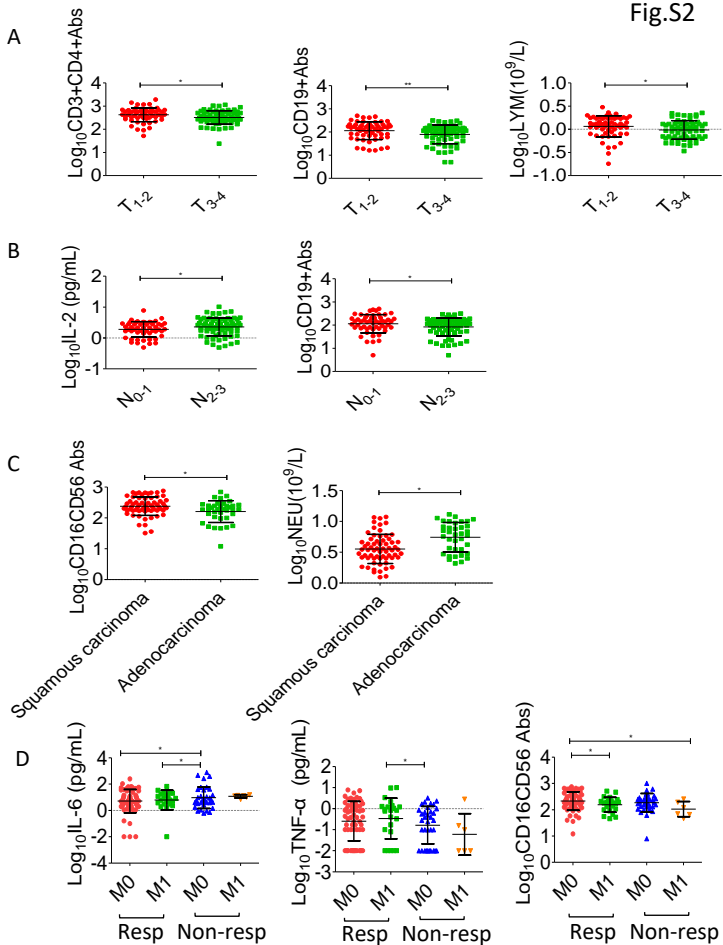

**E**

| Variables | T <sub>1-2</sub> | T <sub>3-4</sub> | <i>p</i> | N <sub>0-1</sub> | N <sub>2-3</sub> | <i>p</i> | M0 | M1 | <i>p</i> |
|-----------|------------------|------------------|----------|------------------|------------------|----------|----|----|----------|
| Male      | 40               | 53               | 0.146    | 40               | 51               | 0.845    | 72 | 22 | 0.124    |
| Female    | 18               | 13               |          | 13               | 18               |          | 24 | 7  |          |

Supplement: Supplementary 2 — Figure S2: analysis of immune cells and cytokines in different stages and differentiations of cancers. (a) Between T1-2 and T3-4 and (b) between N0-1 and N2-3 and (c) between squamous carcinoma and adenocarcinoma of respiratory cancers and (d) between M0 and M1 of respiratory and nonrespiratory cancers. (a) CD3+CD4+ Abs (left), CD19+ Abs (middle), LYM (right). (b) IL-2 (left), CD19+ Abs (right). (c) CD16+CD56+ Abs (left), NEU (right). (d) IL-6 (left), TNF-α (middle), CD16+CD56+ Abs (right). (e) Sex variables in different TNM stages of cancer patients. [file 8669098.f2.pdf]

Fig.S3

M0 vs M1

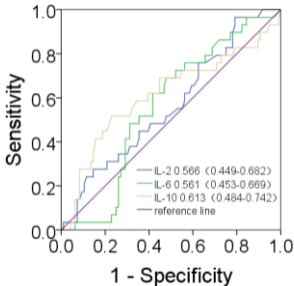

Supplement: Supplementary 3 — Figure S3: ROC curve analysis of immune cells and cytokines between metastatic and nonmetastatic patients. [file 8669098.f3.pdf]
