# Supplementary material for: COVID-19 and Cancer: Discovery of Difference in Clinical Immune Indexes
Source: J Immunol Res. 2021 Oct 18;2021:8669098. doi: 10.1155/2021/8669098 (PMC8546403; doi:10.1155/2021/8669098)
Supplement: Supplementary 4 — Table S1: stages and differentiation types of cancers. [file 8669098.f4.docx]

Table S1 Stages and differentiation types of cancers

| Variables | Cancers (%) |  |
| --- | --- | --- |
|  |  |  |
| Stage |  |  |
| T |  |  |
| T_1-2_ | 58(26.6%) |  |
| T_3-4_ | 66(30.3%) |  |
| Unknown* | 94(43.1%) |  |
| N |  |  |
| N_0-1_ | 53(24.3%) |  |
| N_2-3_ | 69(31.7) |  |
| Unknown* | 96(44%) |  |
| M |  |  |
| M0 | 96(44%) |  |
| M1 | 29(13.3%) |  |
| Unknown* | 93(42.7%) |  |
| Cancer types |  |  |
| Non-Respiratory cancers* |  |  |
| Squamous carcinoma | 46(47.4%) |  |
| Adenocarcinoma | 16(16.5%) |  |
| Others | 35(36.1%) |  |
| Respiratory cancers |  |  |
| Squamous carcinoma | 64(52.9%) |  |
| Adenocarcinoma | 37(30.6%) |  |
| Others* | 20(16.5) |  |

Abbreviations: T, tumor; N, lymph node; M, metastasis; * Analysis of variance was excluded.
